# Electrodeposition of Ni–Mo alloy coatings from choline chloride and propylene glycol deep eutectic solvent plating bath

**DOI:** 10.1038/s41598-022-22007-4

**Published:** 2022-11-02

**Authors:** Anna Niciejewska, Aleeza Ajmal, Mirosława Pawlyta, Marek Marczewski, Juliusz Winiarski

**Affiliations:** 1 Group of Surface Technology, Department of Advanced Material Technologies, Faculty of Chemistry, Wrocław University of Science and Technology, Wybrzeże Wyspiańskiego 27, 50-370 Wrocław, Poland; 2 Materials Research Laboratory, Faculty of Mechanical Engineering, Silesian University of Technology, Konarskiego 18A, 44-100 Gliwice, Poland

**Keywords:** Materials science, Nanoscience and technology

## Abstract

Ni–Mo alloy coatings were deposited on a copper base material from a non-aqueous plating bath based on a deep eutectic solvent (DES) of choline chloride and propylene glycol in a 1:2 molar ratio containing 0.2 mol dm^−3^ NiCl_2_ · 6H_2_O and 0.01 mol dm^−3^ (NH_4_)_6_Mo_7_O_24_·4H_2_O. Uniform and adherent Ni–Mo deposits with a nodular morphology were obtained at all the deposition potentials investigated (from − 0.5 to − 0.9 V vs. Ag). By shifting the potential from − 0.5 to − 0.9 V, the deposition current density increased from − 0.4 to − 1.5 mA cm^−2^ and the overall surface roughness increased. It was also accompanied by an increase in the Mo content from ~ 7 to ~ 13 wt% in the potential range from − 0.5 to − 0.7 V. A further change in the potential from − 0.8 to − 0.9 V caused a decrease in the Mo content to ~ 10 wt% and a deterioration in the quality of the coating. For the most uniform coating, deposited at − 0.6 V and having a thickness of ca. 660 nm, the crystallite size did not exceed 10 nm. With the content of Ni (89 at.%) and Mo (11 at.%), the selected area electron diffraction (SAED) analysis allowed us to identify the cubic phase Ni_3.64_Mo_0.36_. The corrosion resistance of Ni–Mo coatings in 0.05 mol dm^−3^ NaCl solution generally increased during exposure of 18 h, as evidenced by ever higher polarization resistance. Finally, regardless of the applied deposition potential, low corrosion currents (in the range of 0.1–0.3 μA cm^−2^) have been measured for the coatings. EIS revealed that charge transfer resistances were the highest (57–67 kΩ cm^2^) for coatings deposited at − 0.5 V, − 0.6 V and − 0.7 V. Further increase in the deposition potential in the negative direction was unfavorable.

## Introduction

Ni–Mo alloy coatings provide considerable improvements compared to traditional monolithic Ni coatings in terms of their increased hardness^1^, corrosion^2^, wear, and thermal resistance^3^, making them suitable as ecological alternatives to chromium coatings. Electrodeposition of Ni–Mo coatings produces more environmentally harmless wastewater, since molybdenum is a non-toxic metal for the aquatic environment, while the industrial chromium electroplating process still requires the use of carcinogenic Cr^6+^ ions^3,4^. In recent years, a deeper investigation carried out on Ni–Mo alloy coating revealed that these coatings are considered as the electrodes for hydrogen and oxygen evolution reactions^5^.

Electrodeposition is a relatively simple, low cost, and low temperature method suitable for the mass production of Ni–Mo alloy coatings. Almost all Ni–Mo electroplating is performed in aqueous electrolyte baths^3,5,6^. Water provides the most obvious and convenient solvent to formulate a plating electrolyte, since it is readily available, nontoxic, and can easily dissolve many metal salts. However, water-based galvanic baths have some limitations. Under certain conditions, a large amount of hydrogen is released from the water baths. This by-process reduces the deposition efficiency and adversely affects the corrosion resistance of the Ni–Mo coatings obtained. In addition, water baths have a fairly narrow potential window, and they can be reactive toward some metals. In bath compositions, toxic compounds such as cyanides and many organic additives (including stabilizers, complexing compounds, and surfactants) are often present. The multitude of compounds present in the water baths leads to an increased production of wastewater that is difficult to dispose of and to an increased need for the treatment of large amounts of water containing heavy metals^7^.

To avoid the aforementioned disadvantages, the electrodeposition of Ni and its alloys from deep eutectic solvents (DES) have attracted substantial interest in recent years^8^. DES electroplating does not require the use of toxic aqueous precursors, which are present in commonly used Ni, Cr, and Co. coating technologies. This will make it possible to circumvent the legal restrictions related to the use and disposal of carcinogenic electroplating baths^9^. Compared to water-based electrolytes, DESs have some great properties, such as a wider potential window, high conductivity, high solubility of metal salts, good thermal stability, and negligible hydrogen evolution on the electrode, which make them suitable electrolytes for the electrodeposition of Ni–Mo alloys. They also produce nanocrystalline deposits with a low surface roughness compared to aqueous solutions. Additionally, particle suspensions are also stable over a long period with a DES base plating bath because of a combination of increased viscosity and the coulombic screening of the surface charge by the ionic liquid. Therefore, DES does not require the use of surfactants and offers an attractive environment for the electrodeposition of nickel and nickel composite coatings without the need to use organic additives to obtain nanocrystalline material with low surface roughness and sufficient corrosion resistance^10^. This advantage, does not exclude the use of additives in DES-based baths. For instance, information on the use of nicotinic acid^11^ and ethylenediamine^12^ in the ChCl: urea plating bath is available in the literature. These additives influenced the morphology and microstructure of the obtained coatings, and therefore the obtained coatings were characterized by high homogeneity and gloss. The tested compounds formed complexes with Ni^2+^ ions and later adsorbed on the electrode surface. This led to a reduction in nickel particle size and a reduction in deposition current^9^. Composite coatings and alloy coatings obtained from baths based on DES are also becoming more and more popular. Nickel is often used as a matrix. Many composite coatings have been successfully obtained in DES baths: Ni/SiO_2_^13,14^, Ni/TiO_2_^15^, Ni/CeMoOxide^10^, Ni/SiC^16^, Ni/PTFE^17^, Ni/CNTs^18^. The presence of composite additives affects the morphology, roughness, crystallinity, and mechanical properties of the coatings obtained.

The use of deep eutectic solvents enables relatively easy electrodeposition of alloys, even those containing rare earth elements. This creates unique and important opportunities also in industrial solutions^9,19,20^. Research conducted on baths based on DES was carried out to obtain alloy coatings, e.g. Zn–Ni^21^, Ni–Co–Sn^22^, Fe–Ni^23^, Ni–Mo–Cu^24^. Electrodeposition of homogeneous, crack-free and adherent Ni–Mo alloy coatings, even in DES plating baths, is a complex process affected by many factors and their interaction. The composition of the plating bath and plating parameters represent major factors necessary to obtain deposits with desirable composition, microstructure, and other properties. Ni–Mo electrodeposition is sensitive to the type of DES, therefore the second factor is the suitable choice of the DES solvent, among which those based on choline chloride (ChCl) dominate. According to the available literature, ChCl-urea, ChCl-urea-citric acid, and ChCl-ethylene glycol-citric acid solvents produce Ni–Mo coatings with 5–6 wt% Mo^25,26^ that have developed a surface and improved catalytic properties for the hydrogen evolution reaction. The addition of a complexing agent (e.g., citric acid) to the DES bath influences positively the reduction process on the one hand^27^ but, on the other hand, it lowers the electrical conductivity of the bath and undoubtedly increases its viscosity. This is not without significance, for example, for the economics of the process or problems with rinsing the detail out of DES plating bath residues. Ni–Mo alloy coatings obtained from DES-based baths (choline chloride–urea–citric acid) are characterized by greater roughness than their counterparts obtained from water baths. The increase in roughness and the resulting increase in the specific surface area are of great importance in the potential use of Ni–Mo alloy coatings as catalytic materials (e.g., in hydrogen evolution reactions)^28^. Therefore, the scientific goal of this work was to determine the feasibility of electrodeposition of Ni–Mo alloy coatings in a simple DES plating bath using ChCl and propylene glycol (PG) as the hydrogen bond donor, due to its lower harmfulness than ethylene glycol (EG).

To achieve this scientific goal, we first used cyclic voltammetry (CV) to determine the range of potentials at which alloy electrodeposition may occur. With this knowledge in mind, a potentiostatic deposition was used to determine the impact of the reduction potential on the properties of the deposits produced. Their morphology and chemical composition were determined using scanning electron microscopy (SEM) and energy dispersive spectroscopy (EDS). Surface topography was measured by contact profilometry. The structure and morphology of selected Ni–Mo coatings were analyzed by transmission electron microscopy (TEM) with focused ion beam (FIB) preparation. Finally, the corrosion resistance the alloy coatings produced was monitored in a 0.05 mol dm^−3^ solution of NaCl using dc polarization techniques and electrochemical impedance spectroscopy (EIS).

## Experimental

### Materials and methods

Copper disks (grade M1E) with a diameter of 14.9 mm and a geometric area of 4.1 cm^2^ were used as the base material. A series of disks was first manually ground using 1200 grit SiC waterproof paper and a Qpol 250 M1 (QATM) grinding and polishing machine. The disks were then washed in deionized water (DI water) using ultrasonic cleaner to remove abrasive residue, degreased in methanol, and dried with compressed air. The DES solvent was prepared by weighing and mixing choline chloride (‘ChCl’, ≥ 98%, Sigma^®^) with propylene glycol (‘PG’, Merck, EMD Milipore) in a 1:2 molar ratio in the ‘as supplied’ form at 45 °C. After complete dissolution of the reagents and formation of DES (denoted ‘blank DES’), metal salts: 0.2 M NiCl_2_·6H_2_O (Sigma-Aldrich, ReagentPlus^®^) and then 0.01 M (NH_4_)_6_Mo_7_O_24_·4H_2_O (BioUltra, ≥ 99.0%) were added to DES. After overnight mechanical stirring at 45 °C, and after all the ingredients were dissolved, the transparent plating bath was used for electrodeposition. Nickel and molybdenum concentrations have been chosen in the plating bath according to the available literature^27^ and our own preliminary experiments, during which it turned out that at the concentration of ammonium heptamolybdate > 0.02 M there was a precipitation of sparingly soluble compounds in the bath volume. The electroplating kit consisted of a 100 ml electrochemical vessel with a thermostat jacket (Metrohm), two Platinode anodes (grid, 30 mm × 60 mm, Umicore), and one silver quasi-reference electrode (99.9% Ag) mounted in a polypropylene lid and an immersion circulator SC100 (Thermo Scientific™) with external circulation to maintain a stable temperature of the DES bath. Electrodeposition was performed at a constant potential (from − 0.5 to − 0.9 V vs. Ag) for 60 min at 50 °C. The conductivity of the bath at the deposition temperature was measured using a CC-551 conductivity meter (Elmetron) and it was 5.8 mS cm^−1^. After that, the samples were thoroughly rinsed first in DI water and then in methanol, dried in hot air, and stored until needed. The deposition rate was determined based on the coating thickness determined by the transmission electron microscope (TEM) and the deposition time (60 min) and was approximately 0.65–0.68 µm h^−1^ at a potential of − 0.6 V.

### Research techniques

Electrodeposition parameters were determined by cyclic voltammetry (CV). The system consisting of Reference 3000 potentiostat (Gamry) and RDE-2 rotating disk electrode (Metrohm) at a rotational speed of 700 rpm was used. Measurements were made in a 100 ml electrochemical cell with a water jacket (Metrohm) using RDE Pt tip (3 mm diameter), the Pt counter electrode, and the Ag quasi-reference electrode. The potentiodynamic scan started from the open circuit potential (E_OC_) to − 2.0 V vs. Ag, then to + 1.5 vs. Ag and finally, was terminated at E_OC_. It has been applied a scan rate of 20 mV s^–1^.

Surface morphology was analyzed by Quanta 250 scanning electron microscope (FEI) at 15 kV in a secondary electron mode (SE). Energy dispersive spectroscopy (EDS) was used to determine the (semiquantitative) chemical composition of the samples (15 kV, area 200 μm × 200 μm). The structure of the deposits was analyzed by a transmission electron microscope –S/TEM Titan 80–300 (FEI) operating at 300 kV. For the analysis of the chemical composition, the energy dispersion spectrometer (EDS) and the energy filter, Gatan imaging filter (Gatan Tridiem 863) were used. The coatings lamella for the TEM analyzes was prepared using the focused ion beam (FIB) technique using the SEM/Ga-FIB Helios NanoLab™ 600i dual-beam microscope (FEI).

The topography of the coatings surfaces was analyzed using the contact profilometry method. For this purpose, a Bruker DektakXT profilometer was used, along with dedicated software, Vision64. Measurements were made according to PN-EN ISO 4287: 1999 and PN-EN ISO 11562: 1998, which define the methodology for measuring the parameters of the sample profile. A stylus with a tip radius of 2.5 μm was used for the analysis, subjected to a load of 3 mg. The scanning speed was 500 μm min^−1^, and the scan was carried out perpendicular to the grinding lines.

The corrosion resistance of Ni–Mo coatings was measured in a 0.05 mol dm^−3^ deaerated solution of NaCl by electrochemical impedance spectroscopy (EIS), linear polarization resistance (LPR), and polarization curves, in the sequence described below. These experiments were carried out in a 400 ml corrosion cell (Metrohm) connected to a Reference 600 potentiostat (Gamry) in a three-electrode setup consisting of: the working electrode (with a geometric area of 1 cm^2^), the counter electrode (316 steel rod with a geometric area of 4 cm^2^) and the Ag|AgCl (3 M KCl) reference electrode (Metrohm) with a Luggin capillary. LPR was measured every hour by sweeping the potential from − 15 to + 15 mV vs. E_OC_ with a scan rate 1 mV s^−1^. Impedance spectra were acquired at the open circuit potential (E_OC_) after 18 h of exposure of the coatings in NaCl solution. The frequency range started from 100 kHz to 1 mHz with 10 pts/dec. resolution and excitation of the 5 mV (rms) ac signal. Finally, potentiodynamic polarization curves were recorded after ~ 24 h of exposure from − 0.1 V to + 0.1 V vs. E_OC_ with a scan rate of 0.167 mV s^−1^. Fitting of the impedance spectra, polarization curve treatment, and determination of the polarization resistance were done using EchemAnalyst (Gamry) software.

## Results and discussion

### Cyclic voltammetry and potentiostatic deposition

**Figure 1:**
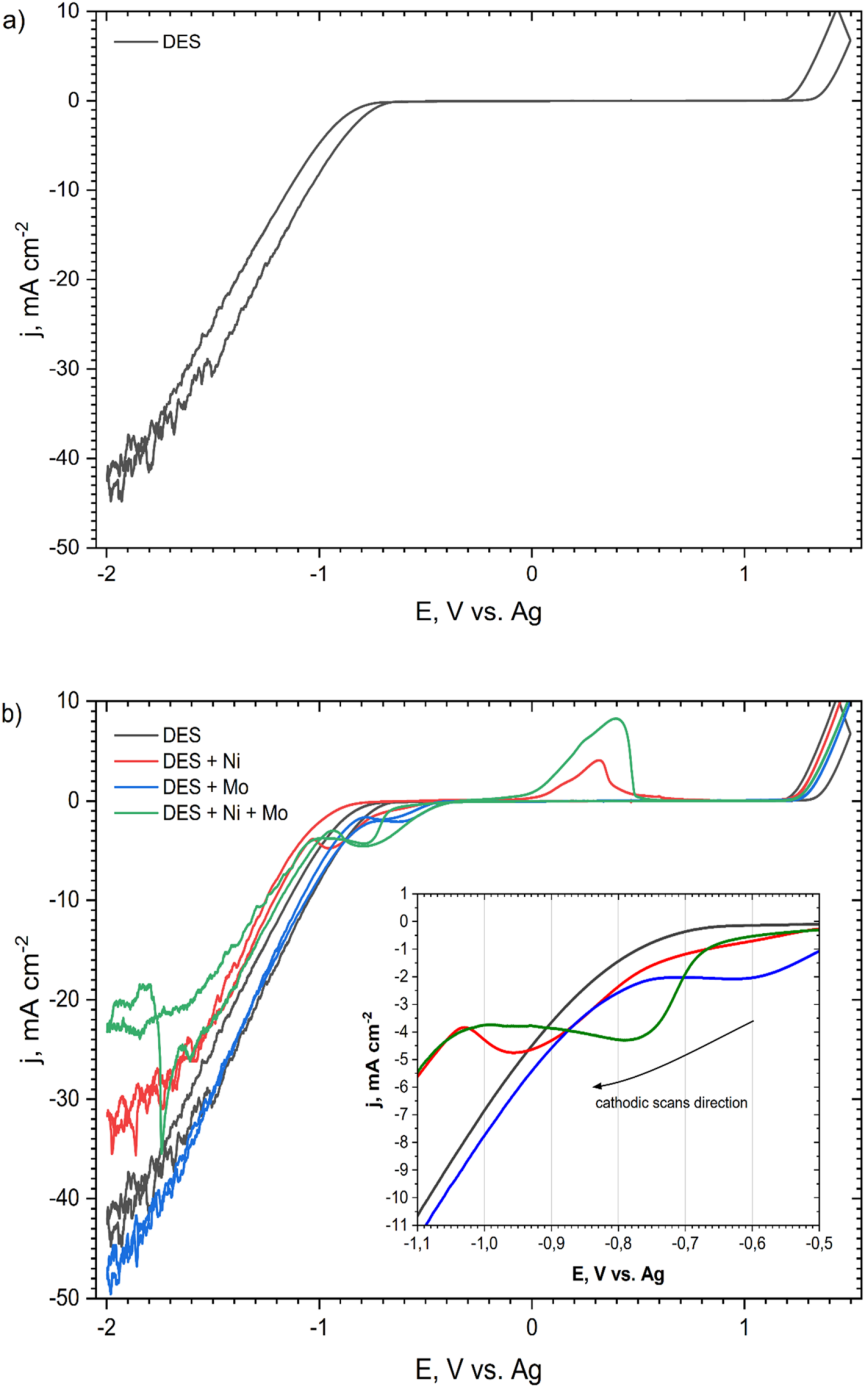
CV curves recorded on the Pt rotating disk electrode at 50 °C with a scan rate of 20 mV s^–1^ and a rotational speed of 700 rpm in: blank ChCl:PG 1:2 (denoted as ‘DES’) (**a**,**b**); ChCl:PG 1:2 + 0.2 mol dm^−3^ NiCl_2_ (denoted as ‘DES + Ni’) (**b**); ChCl:PG 1:2 + 0.01 mol dm^−3^ (NH_4_)_6_Mo_7_O_24_ (denoted as ‘DES + Mo’) (**b**) and ChCl:PG 1:2 + 0.2 mol dm^−3^ NiCl_2_ + 0.01 mol dm^−3^ (NH_4_)_6_Mo_7_O_24_ (denoted as ‘DES + Ni + Mo’) (**b**).

CV curves were recorded in DES plating baths to determine reduction and oxidation potentials. Figure 1 presents the current–potential characteristics recorded for: blank DES (without Ni and Mo compounds; Fig. 1a), DES + 0.2 mol dm^−3^ NiCl_2_, DES + 0.01 mol dm^−3^ (NH_4_)_6_Mo_7_O_24_ and DES + 0.2 mol dm^−3^ NiCl_2_ + 0.01 mol dm^−3^ (NH_4_)_6_Mo_7_O_24_ (Fig. 1b). The curves show how the addition of individual bath components influenced the reduction potential. They were recorded in the potential range of − 2.0 V to + 1.5 V. In the inset in the graph (Fig. 1b), only parts of the polarization curves in the cathodic direction are shown. For blank DES, no peak is visible. Fluctuations in the intensity of currents visible at the lowest potentials (approx. − 1.5 to − 2.0 V for all baths) arise as a result of a side reaction, most likely reduction of hydrogen and partial blocking of the electrode surface by gas bubbles. The cathodic shoulder for the DES + Ni bath is visible in the potential range of approx. − 0.7 to − 1.0 V. For the DES + Mo bath this cathodic shoulder appears at potentials of approx. − 0.5 to − 0.7 V and for the DES + Ni + Mo bath a cathodic plateau is evidenced between − 0.6 to − 0.9 V. The feature that distinguishes the DES + Ni + Mo curve from the others is the presence of a nucleation loop at a potential of approx. − 1.8 V. For the DES + Ni bath, the anodic peak is also visible, which might be related to the oxidation of the metallic layer formed during the cathodic scan. Such a peak is not visible in the course of the DES + Mo bath curve. This may indicate that the reduction does not create a permanent, solid product, or adhesive layer on the electrode. In the case of DES + Ni + Mo baths, the cathodic peak is present in the potential range of approx. − 0.7 to − 0.9 V. These are the average values of the values obtained for the DES + Ni and DES + Mo baths. The DES + Ni + Mo curve also shows an anodic peak, the top of which is characterized by a current value that is approximately twice that of the DES + Ni bath. This proves that a higher charge is involved in the oxidation reactions and a higher mass of the oxidized coating. S. Costovici et al. showed quite similar CV curves for electrolytes containing Ni^2+^ and Mo^6+^ ions in choline chloride-urea-citric acid mixtures. In this case, the co-reduction potential of Ni^2+^ and Mo^6+^ species was located at − 0.75 to − 1.25 V. The anodic peak was located at a similar potential, but it was smaller than the anodic peak from baths containing only Ni^2+^ ions^27^.

**Figure 2:**
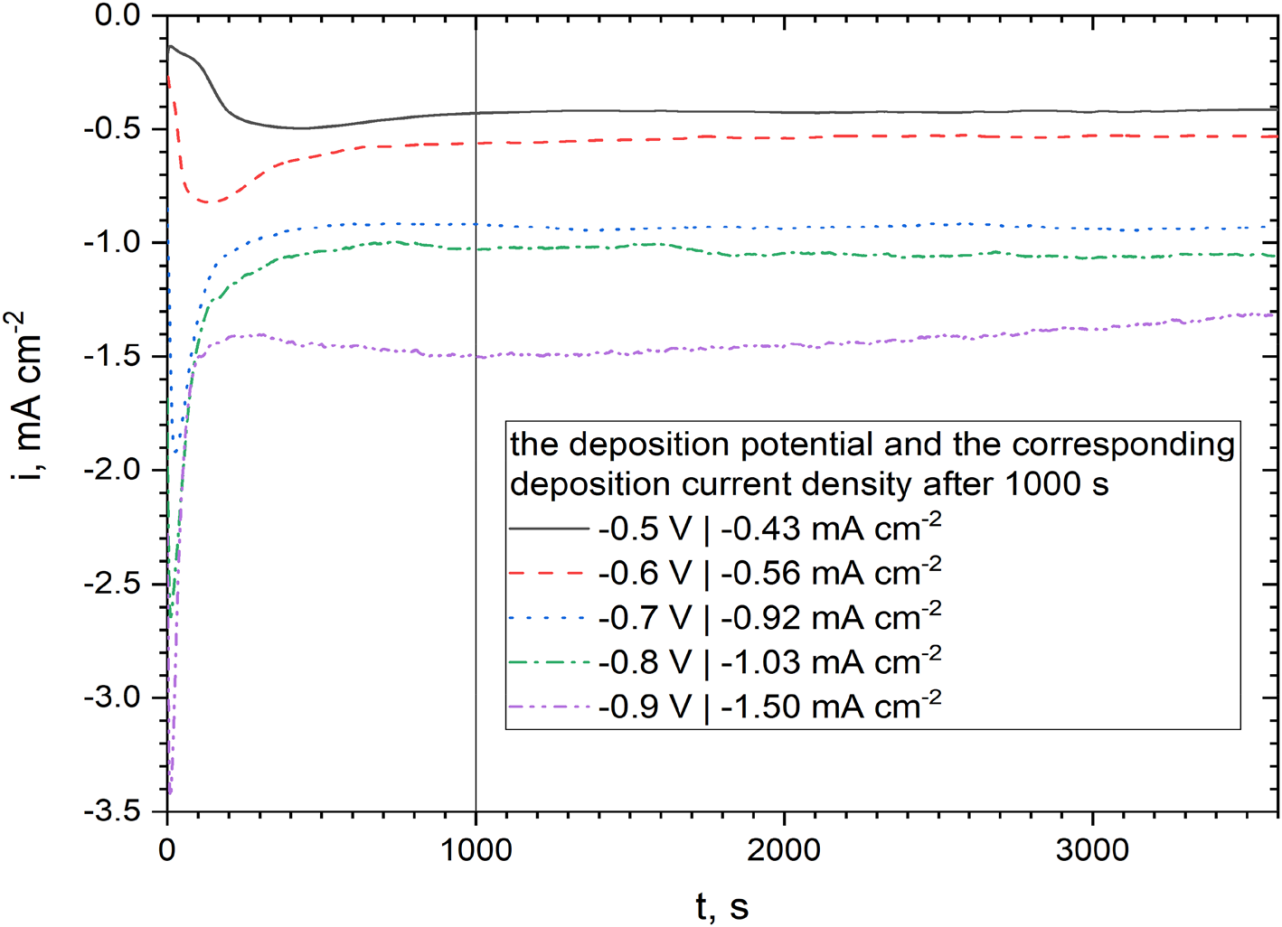
Current density vs. time dependences recorded on Cu base material during 1 h electrodeposition of Ni–Mo coatings at potentials of − 0.5 to − 0.9 V vs. Ag at 50 °C in the plating bath composed of DES + 0.2 mol dm^−3^ NiCl_2_ + 0.01 mol dm^−3^ (NH_4_)_6_Mo_7_O_24_.

Based on the cathodic curve for the bath DES + 0.2 mol dm^−3^ NiCl_2_ + 0.01 mol dm^−3^ (NH_4_)_6_Mo_7_O_24_ bath (Fig. 1), the potential range of − 0.5 to − 0.9 V vs. Ag was selected, where a series of samples were deposited. The evolution of the measured current density against the deposition time at a constant applied potential is shown in Fig. 2. As the deposition potential is more electronegative, an increasingly intense and narrower ‘peak’ of the cathode current is observed within the first second of polarization. Its presence probably indicates the formation of a double layer at this stage of coating nucleation. The increasing intensity of the peak may result from the ever-higher rate of nucleation and changes in the morphology and surface chemistry. The decrease in the cathode current after this stage may in turn result from diffusion limitations (no mixing of the bath) or possible blocking of the cathode surface with a layer of products of incomplete reduction of metal ions. After about 1000 s, the deposition current density for all samples becomes almost constant. A similar tendency of the dependence of the cathode current density vs. electrodeposition time was previously observed in the literature^29^.

### Morphology, topography, and composition of the coatings

**Figure 3:**
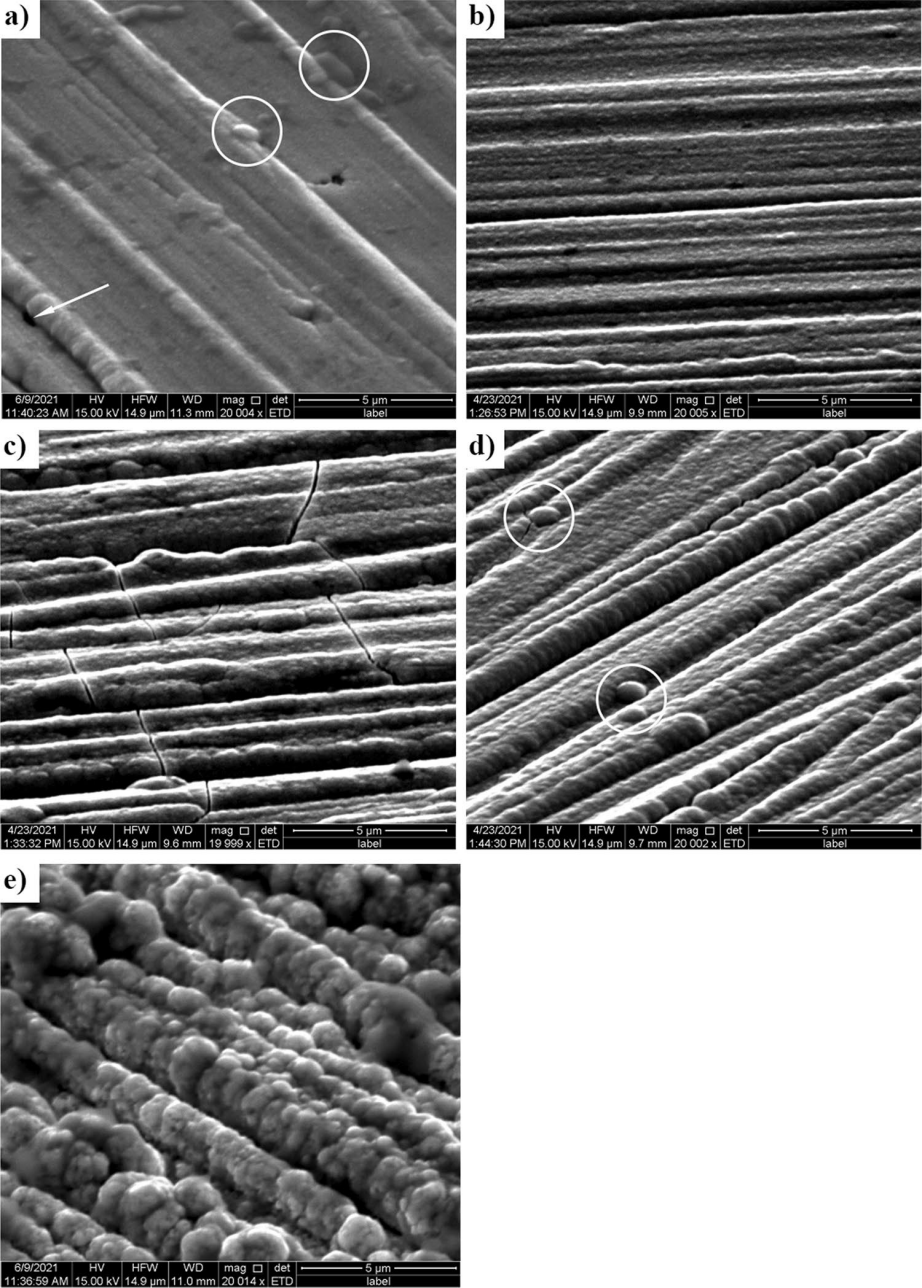
Electron microscope micrographs of the Ni–Mo alloy coatings deposited from DES + 0.2 mol dm^−3^ NiCl_2_ + 0.01 mol dm^−3^ (NH_4_)_6_Mo_7_O_24_ at: − 0.5 (**a**), − 0.6 (**b**), − 0.7 (**c**), − 0.8 (**d**) and − 0.9 V vs. Ag (**e**) for 60 min at 50 °C.

Analysis of the electron microscope microphotographs (Fig. 3) allowed for a preliminary assessment of the quality of the produced coatings. It could be stated that the obtained coatings are rather thin and evenly distributed because the photos still show grinding lines made during the mechanical surface treatment. The most visually attractive coating, with the lowest visible graininess, was obtained at a potential of − 0.6 V vs. Ag (Fig. 3b). From the analysis of the photos, it can also be assumed that the deposited coatings have a more developed surface with increasing (toward negative values) potential of the deposition. It could also be connected with the increase in the thickness of the coatings. For example, in Fig. 3c cracks in the coating can be seen, which were caused by its growth, and in Fig. 3d,e one can see a significant increase in coating graininess, as the agglomerates themselves tend to settle in the discontinuities of the substrate. This could have happened by increasing the deposition rate of the coatings toward a more negative reduction potential, as shown in Fig. 2, which also proves a decrease in the quality of the produced coatings. Single precipitations can also be observed in the sample with Ni–Mo coating deposited at the lowest voltage, equal to − 0.5 V (Fig. 3a). When comparing the obtained coatings to the results obtained by other research groups, one can notice the similarity of structures on the surface^27,30^. In the case of Ni–Mo coatings deposited on a steel substrate from an aqueous plating bath of salts of these metals^30^, a tendency to cracking of these deposits can be observed, as well as the formation of characteristically structured agglomerates of various sizes, depending on the process parameters. Successful deposition of Ni–Mo coatings was also carried out in eutectic solvents, this time on a copper substrate^27^, with similar process parameters. In this work, the coatings shown in Fig. 3 are less regular, which may be caused, for example, by the use of a different eutectic solvent or the preparation of the surface of the substrate.

**Figure 4:**
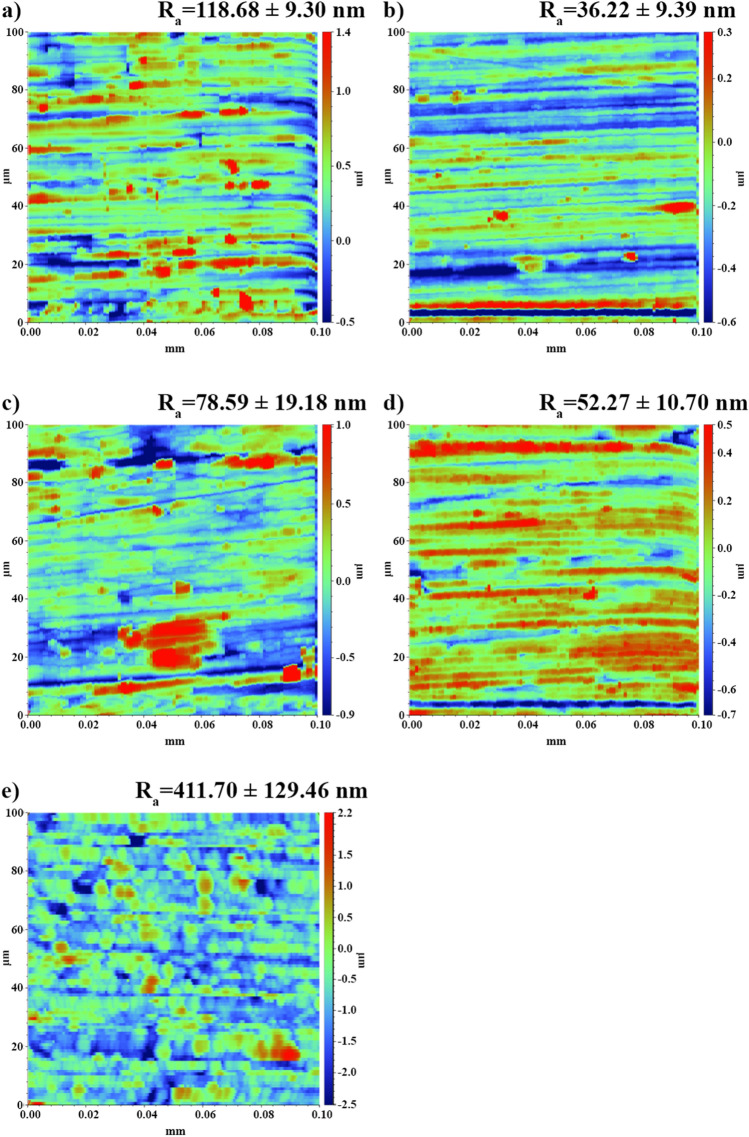
Topographic maps and the arithmetic average of the roughness profile (R_a_) of the Ni–Mo alloy coatings deposited from DES + 0.2 mol dm^−3^ NiCl_2_ + 0.01 mol dm^−3^ (NH_4_)_6_Mo_7_O_24_ at − 0.5 (**a**), − 0.6 (**b**), − 0.7 (**c**), − 0.8 (**d**) and − 0.9 V vs. Ag (**e**) for 60 min at 50 °C.

The research on the topography of the samples confirms that the roughness of the samples increased along with the increase (toward negative values) of the deposition potential (Fig. 4). By comparing the results from stylus profilometry to the microscopic photos obtained from the samples, one can confirm a decrease in the quality of the electrodeposited coatings, which is manifested by the presence of larger agglomerates on the surface of the samples. For example, the coating deposited at − 0.9 V vs. Ag showed the highest surface roughness, R_a_ = 450 nm (Fig. 4e), and high graininess of the sample was observed in the samples deposited at − 0.7 V vs. Ag and − 0.8 V vs. Ag (Fig. 4c,d). The sample with a coating deposited at − 0.5 V vs. Ag (Fig. 4a) was characterized by high roughness, which, in combination with the visible topographic map of the surface, indicates uneven deposition of the applied coating, which was not observed in the microscopic photo. The lowest surface roughness, R_a_ = 36 nm, was obtained in the sample with a coating deposited at − 0.6 V vs. Ag (Fig. 4b). Due to the desired visual properties and topographic parameters of the coating, it was decided that it would be used for further studies of the coating properties.

**Figure 5:**
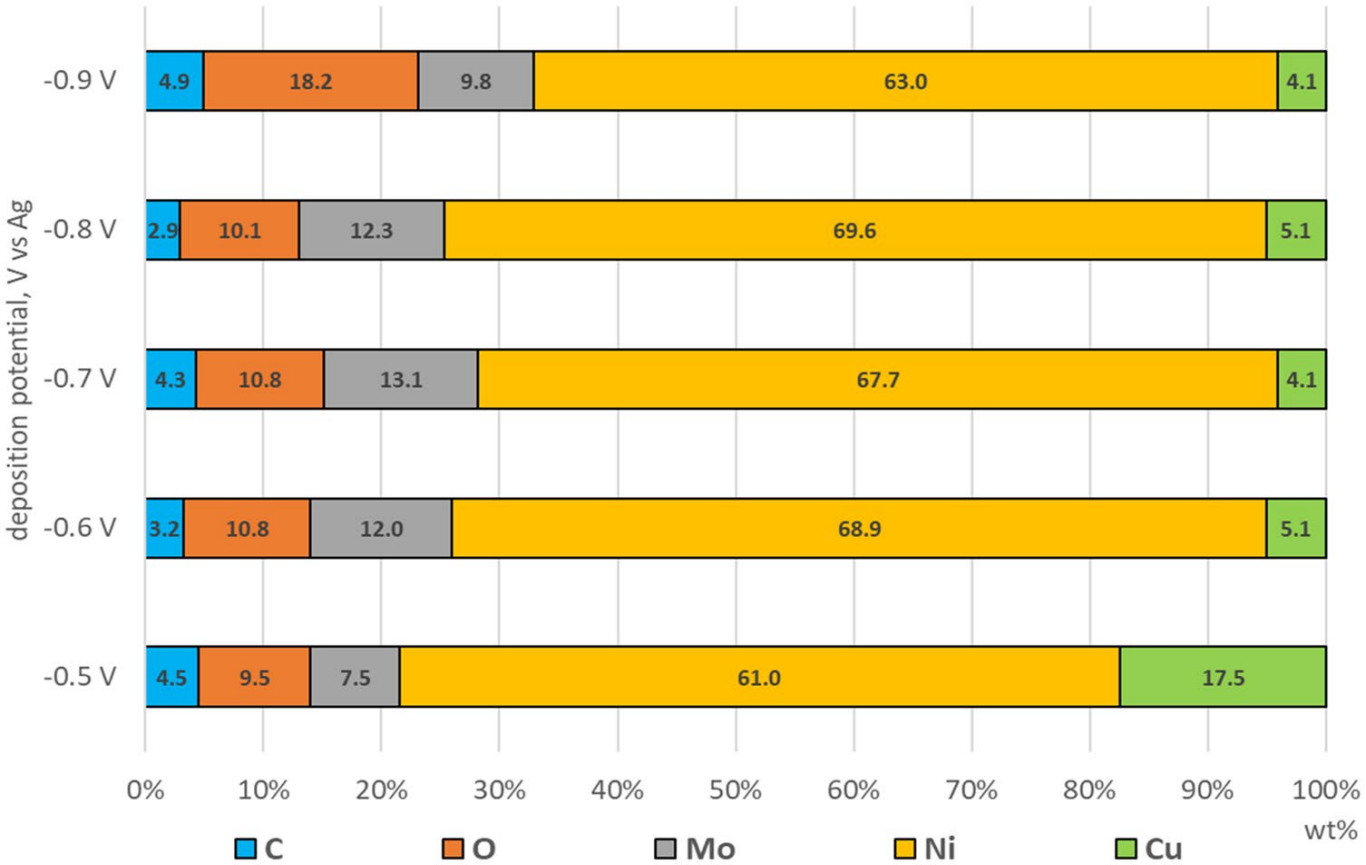
Results of EDS analysis performed on samples with Ni–Mo alloy coatings deposited from DES + 0.2 mol dm^−3^ NiCl_2_ + 0.01 mol dm^−3^ (NH_4_)_6_Mo_7_O_24_ at − 0.5, − 0.6, − 0.7, − 0.8, − 0.9 V vs. Ag for 60 min at 50 °C.

Semi-quantitative EDS analysis allowed one to estimate the influence of the deposition potential on the chemical composition of samples with Ni–Mo coatings (Fig. 5). It was shown that as the potential changed from − 0.5 to − 0.7 V, the molybdenum content increased. With a further shift of the deposition potential to − 0.8 and − 0.9 V, the Mo content decreased. Wasekar et al. observed a similar increase and decrease in Mo content when changing the current density. The probable cause of this phenomenon is the increase in the rate of intermediate reactions, with increasing current density, which inhibits the reduction of Mo^31^.

The oxygen content increased with decreasing deposition potential. The − 0.9 V coating (approx. 18 wt%) has the highest oxygen content. The increase in oxygen content may indicate the formation of metal oxides on the surface. Alternatively, oxygen can be incorporated into the deposits from some residues of organic components of the plating bath. The copper present in the EDS analysis comes from the base metal. A large amount of copper in the analysis of the coating of − 0.5 (approximately 18 wt%) proves that the thickness of this coating is much lower than in the case of other samples. Based on the TEM analysis (Fig. 7a), the thickness of the − 0.6 V coating was 660 nm (approx. 5 wt% Cu). The presence of carbon in the analysis is a common phenomenon and may come from the organic components of the bath, some of which may have been incorporated or adsorbed during electrodeposition.

### Structure of the coatings. TEM analysis

**Figure 6:**
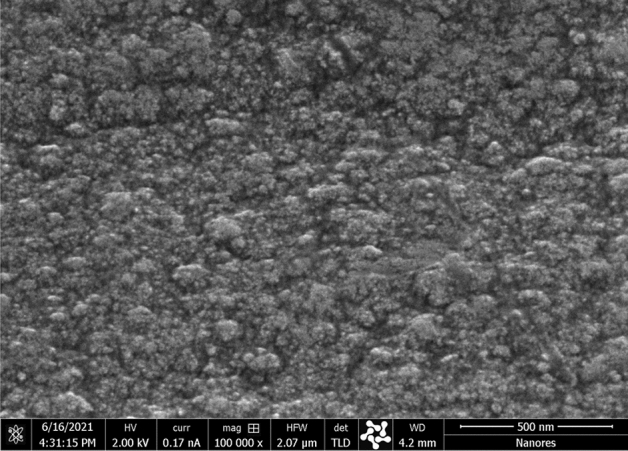
HR-SEM micrograph of the surface of Ni–Mo alloy coating deposited from DES + 0.2 mol dm^−3^ NiCl_2_ + 0.01 mol dm^−3^ (NH_4_)_6_Mo_7_O_24_ at − 0.6 V vs. Ag for 60 min at 50 °C.

The low thickness of the deposited Ni–Mo coatings was the reason why conventional X-ray diffraction (XRD) analysis proved ineffective in determining their structure. Ultimately, it was decided to perform a TEM structural analysis for Ni–Mo coating deposited at − 0.6 V vs Ag because it was characterized by the best quality among all samples investigated. The homogeneous surface, free of visible defects, where the lamella was taken for TEM analysis, is shown in Fig. 6.

**Figure 7:**
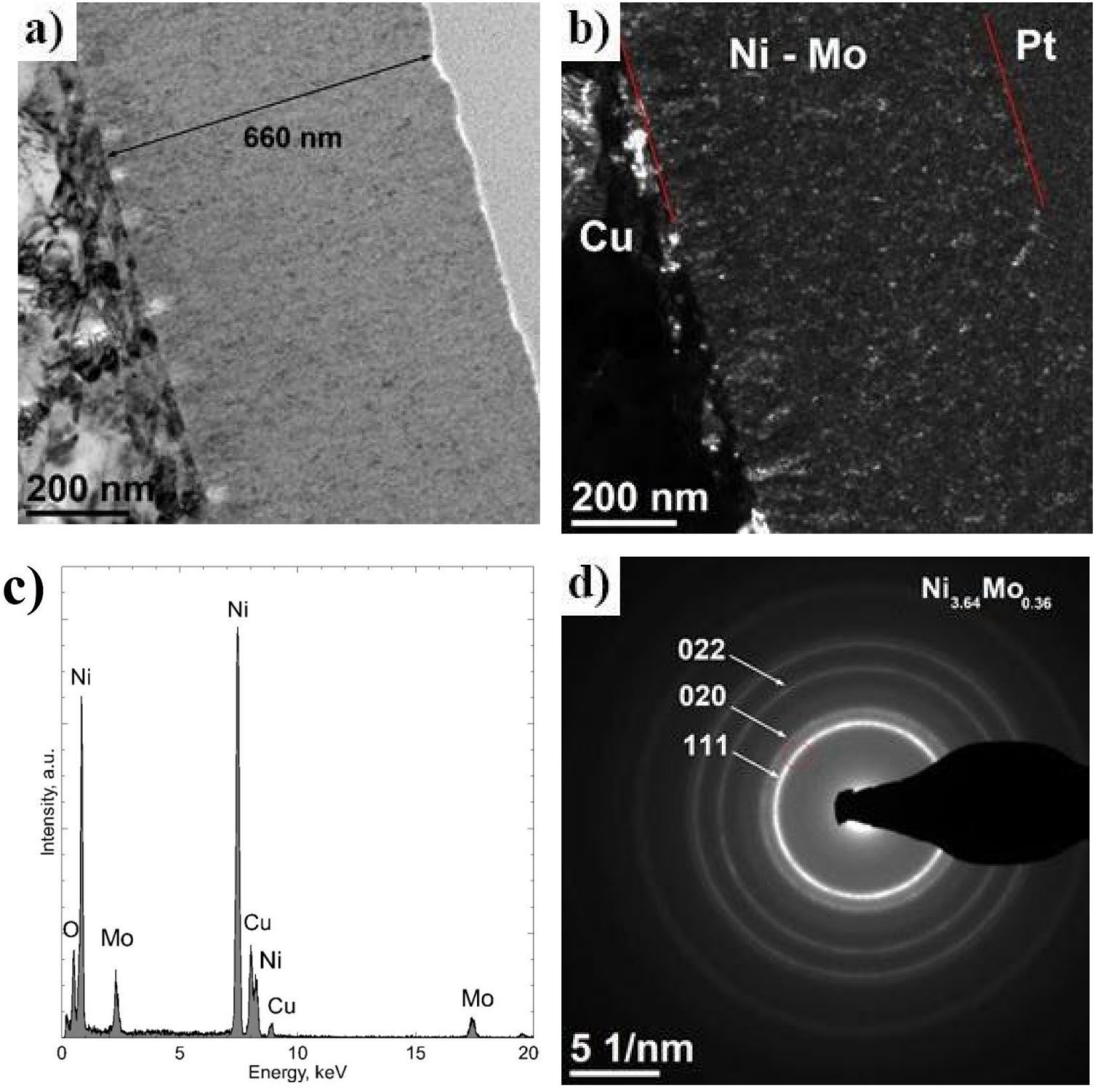
Cross section of the Ni–Mo coating deposited at − 0.6 V vs. Ag for 1 h at 50 °C: TEM BF image (**a**), DF TEM image (**b**), EDS analysis result in the area of coating (**c**), SAED, the red color (circle) marks the position of the objective aperture used to obtain the DF (**d**).

TEM microscopic analysis showed, first of all, that the Ni–Mo coating was characterized by a relatively uniform thickness, ca. 660 nm (Fig. 7a). It also revealed its homogeneous nanocrystalline structure. The crystallite size visible in the dark-field image did not exceed 10 nm (Fig. 7b). Analysis of the chemical composition using the characteristic X-ray energy dispersion technique showed the presence of Ni in the coating (89 at.%) and Mo (11 at.%) (Fig. 7c). Cu and O were not included in the quantitative analysis. Diffraction with the use of a selective shutter (SAED) made in the area of the coating of the deposited Ni–Mo alloy allowed to identifying the cubic phase (space group Fm-3m) Ni_3.64_Mo_0.36_ (JCPDS 96-152-2538)^32^. The rays of the circles visible in the diffraction image correspond successively to the distances between the planes with indicators (111), (020), and (022) of this phase (Fig. 7d). The formation of various intermetallic phases during the electrodeposition process of Ni–Mo coatings is known in the literature. Temem et al., by changing the deposition current density, obtained coatings with different intermetallic phases^33^. It was also related to the change in Mo content in the coatings. The phases obtained are MoNi_4_, Mo_1.24_Ni_0.76_, and Ni_3_Mo. The intermetallic phases in the coatings were also obtained by annealing the obtained coatings. Lima-Neto et al. thus obtained coatings containing the phases: Ni_4_Mo and NiMo^34^.

**Figure 8:**
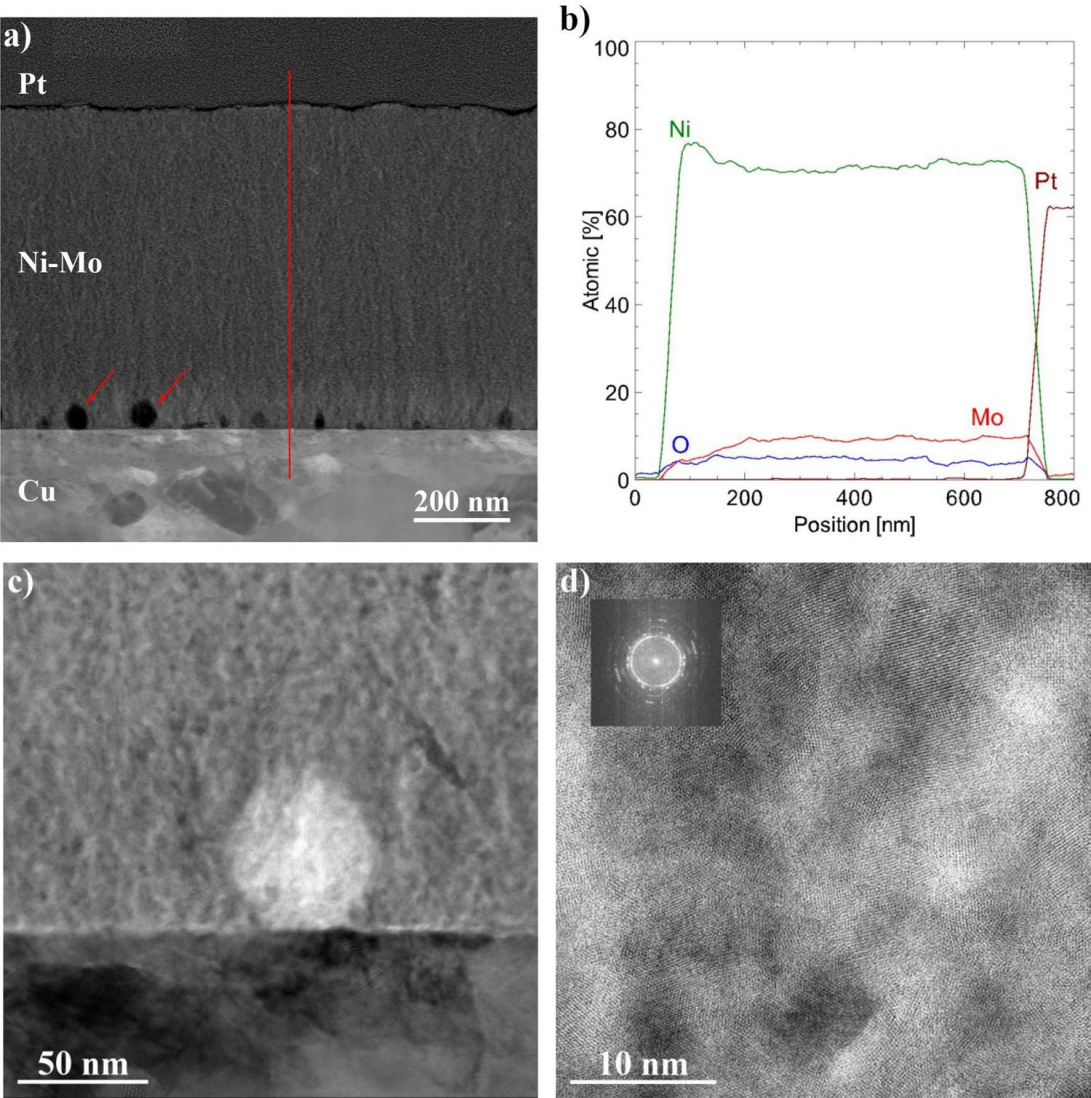
Cross-section of the Ni–Mo coating deposited at − 0.6 V vs. Ag for 1 h at 50 °C: STEM HAADF image (**a**) profile analysis of changes in chemical composition along the red line (**b**), STEM-BF (**c**), HR STEM-BF along with the designated FFT (**d**).

Figure 8a shows a cross-sectional image of a coating obtained in STEM mode with an HAADF detector. The HAADF image reveals the chemical contrast (Z-contrast, where the intensity of the recorded signal is proportional to the atomic number of the elements present in the sample). Two important facts can be seen from it. First, the coating is homogeneous in terms of its chemical composition (there is no contrast change in the coating area). This is also confirmed by the profile analysis (Fig. 8b). Only at the interface of the copper base material/Ni–Mo coating can the nickel content be seen that it was initially higher and the molybdenum content lower than their content in the coating volume. Indeed, Ni has an important role in the induced co-deposition of Mo. It is related to the classical definition of co-induced deposition. Another interesting observation was the presence in this area of the coating of nanometric spherical objects, marked with red arrows in Fig. 8a. They are dark in the HAADF image, indicating that they are less dense areas than the coating, or that they might be voids (pores). Their diameter is around 10–40 nm (Fig. 8c). The EDS spot analysis did not show that these features were enriched with elements other than those present in the Ni–Mo coating. The crystal structure is visible in high-resolution images (Fig. 8d); however, the FFT analysis (inset in the upper left corner) did not show additional interplanar distances compared to the coating. Thus, it can be assumed that the observed objects are pores, and that the visible crystal structure comes from the surrounding Ni–Mo coating.

### Corrosion resistance of the coatings

**Figure 9:**
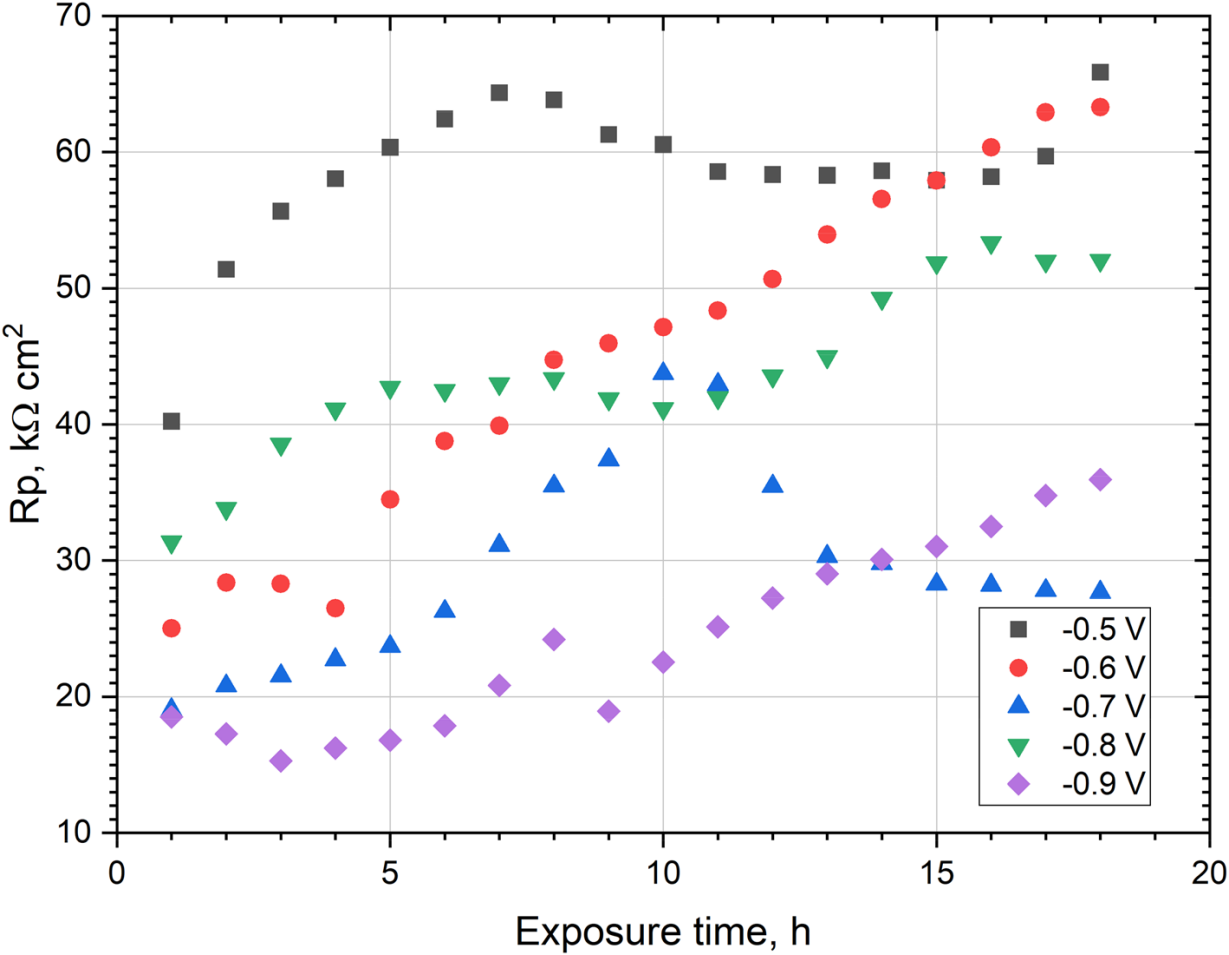
Polarization resistance (R_p_) recorded during 18 h of exposure in 0.05 mol dm^−3^ deaerated NaCl solution of Ni–Mo coatings.

LPR testing was performed in a 0.05 mol dm^−3^ deaerated NaCl solution, while the measurement was performed every hour during the 18-h exposure of the coating. Figure 9 shows the effect of the exposure time in solution on R_p_. R_p_ values were also compared with the Mo content (Fig. 5) in the coatings obtained. In short exposure times, the highest R_p_ values were found for the coating deposited at − 0.5 V vs. Ag. It contained ~ 7.5 wt% Mo, which is the least of all the coatings obtained. The average values of R_p_, in the first hours, reached the coating obtained at potentials − 0.8 V and − 0.6 V. They were characterized by a similar Mo content, approx. 12 wt%.

The lowest values of R_p_ were characteristic for the coatings deposited at potentials of − 0.7 V and − 0.9 V vs. Ag. The Ni–Mo deposit obtained at − 0.7 V contains the most Mo—approx. 13 wt%. Unfortunately, the high molybdenum content could cause microcracks formation in this coating, visible in the SEM photo (Fig. 3c). The coating deposited at − 0.9 V vs. Ag contains the most oxygen and has the most developed and uneven surface of all coatings. During the following hours of exposure in the solution, the R_p_ of all coatings increased. A possible explanation for this phenomenon is the formation of a passive layer/layer of corrosion products on the surface of these coatings, which was previously indicated by Laszczyńska et al.^7^. One of the exceptions was the − 0.7 V coating, for which R_p_ started to decrease after 10 h. The cause of this deterioration in corrosion resistance may be the previously mentioned cracks. The greatest increase in R_p_ after 18 h of exposure in solution was observed for the − 0.6 V coating (an increase of approx. 37 kΩ cm^2^).

**Figure 10:**
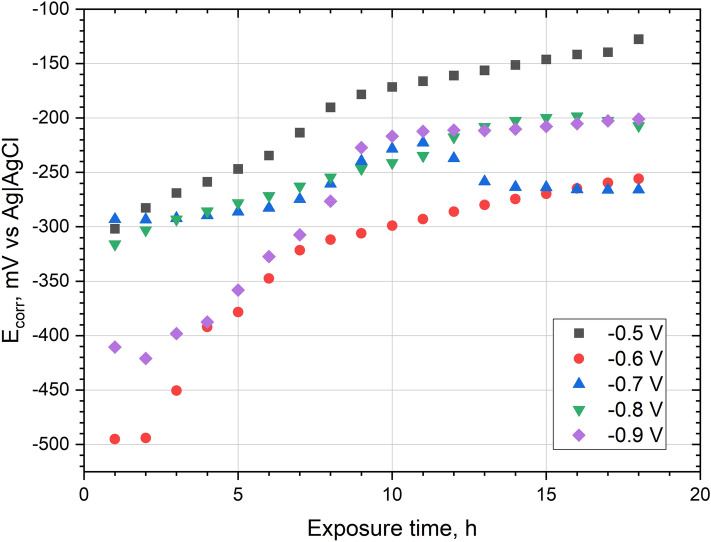
Corrosion potential (E_corr_) recorded during 18 h of exposure in 0.05 mol dm^−3^ deaerated NaCl solution of Ni–Mo coatings.

During 18 h of exposure in 0.05 mol dm^−3^ deaerated NaCl solution, the corrosion potential (E_corr_) of all Ni–Mo coatings was increased (Fig. 10). The highest E_corr_ values are achieved for the coating deposited at − 0.5 V vs Ag, and the lowest for the coating obtained at − 0.6 V vs Ag. The difference between E_corr_ of the coatings may be affected by the condition of the surface, e.g. passive layer, oxides, inhomogeneities, and also base material. In the E_corr_ graph as a function of time for the coating deposited at potential − 0.7 V, some local maximum is visible. After about 10 h the E_corr_ values decrease. A similar maximum is observed in the plot of R_p_ (Fig. 9). The increase in E_corr_ (as well as the increase in R_p_) may be caused by the formation of a layer of corrosion products on the surface of the coatings during exposure in the chloride environment.

**Figure 11:**
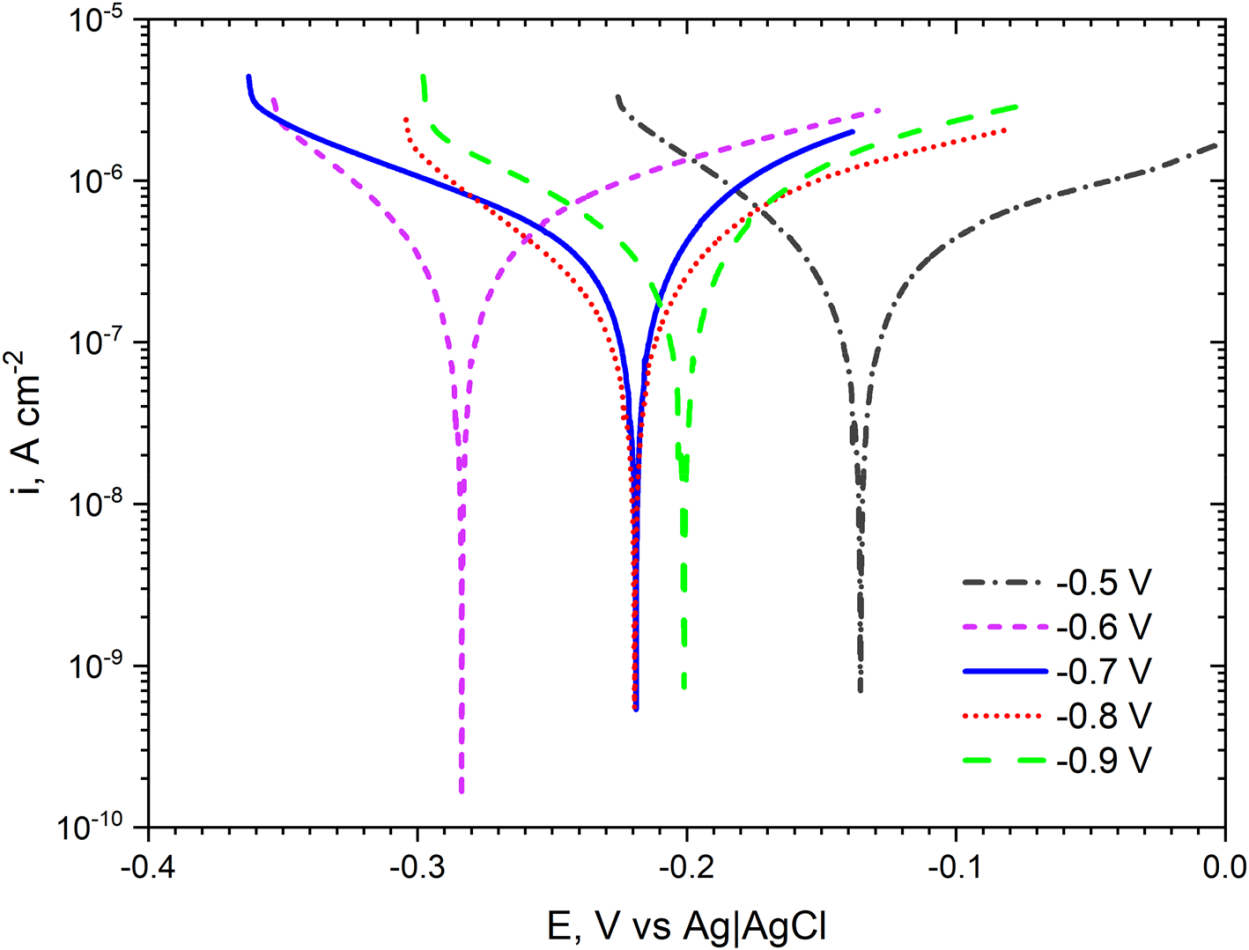
Potentiodynamic polarization curves recorded after 24 h of exposure in 0.05 mol dm^−3^ deaerated NaCl solution of Ni–Mo coatings.

To better illustrate the corrosion phenomena, potentiodynamic curves have been determined after 24 h of coatings exposure in 0.05 mol dm^−3^ NaCl solution (Fig. 11). The coating deposited at − 0.5 V had the highest cathode-to-anode transition potential. For the remaining coatings, this potential was more electronegative and the greatest difference between the potentials obtained was 0.148 V (coatings deposited at − 0.5 V and − 0.6 V). The coating deposited at − 0.6 V had the lowest potential. For the coatings deposited at − 0.7 V, − 0.8 V, − 0.9 V, these values were the closest to each other. All the resulting curves have a similar shape. The shape of the anode curves indicates the oxidation processes that take place on the surface. It seems that there are no diffusion and passive processes on the surface of the coatings. Guettaf Temam et al. observed similar, that as the deposition current density increased, the corrosion potential shifted toward more negative values for Ni–Mo coatings in 0.6 mol dm^−3^ solution^33^.

Table 1 lists the parameters that characterize the curves in Fig. 11. The corrosion current was highest for the coating deposited at − 0.7 V. The values of the corrosion currents obtained are consistent with those determined based on the measurement of the polarization resistance (Fig. 9). The determined slopes of the anode and cathode (βc, βa) curves are also typical (they are within the range 60–120 mV dec^−1^).

**Table 1:** Corrosion parameters (E_corr_, i_corr_, β_c_, β_a_) determined from the potentiodynamic curves of the Ni–Mo alloy coatings recorded after 24 h of exposure of the samples in 0.05 mol dm^−3^ NaCl solution.

Deposition potential (E, V vs Ag)	E_corr_ [V vs. Ag|AgCl]	i_corr_ [μA cm^−2^]	β_c_ [mV dec^−1^]	β_a_ [mV dec^−1^]
− 0.5 V	− 0.136	0.21	78	108
− 0.6 V	− 0.284	0.23	71	80
− 0.7 V	− 0.219	0.29	129	86
− 0.8 V	− 0.219	0.13	76	63
− 0.9 V	− 0.201	0.24	93	71

**Figure 12:**
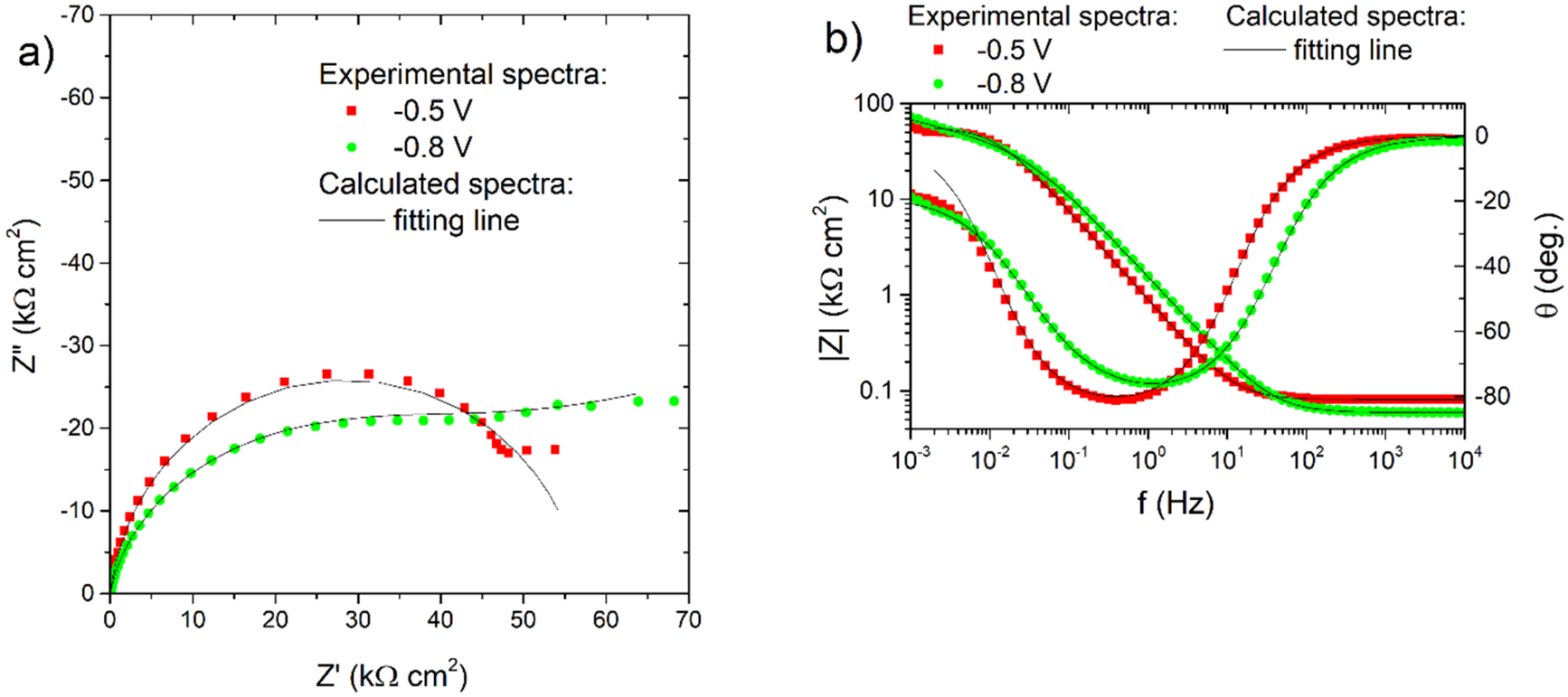
Nyquist (**a**) and Bode (**b**) graphs for the impedance spectra of selected Ni–Mo coatings recorded at E_OC_ after 18 h of exposure of the samples in 0.05 mol dm^−3^ NaCl solution together with fitting lines.

Finally, electrochemical impedance spectroscopy (EIS) was used to characterize the corrosion process of the obtained Ni–Mo coatings in sodium chloride solution. A cursory and preliminary comparative analysis of the spectra showed no clear relationship (direction of change) between the electrodeposition potential and the corrosion resistance of Ni–Mo coatings. Therefore, only selected impedance spectra recorded after 18 h of exposure of the coatings in 0.05 mol dm^−3^ NaCl solution were shown in Fig. 12 both in the Nyquist and Bode representations. The shape of the spectra for the sample ‘− 0.5 V’ in the Nyquist plot (Fig. 12a) is similar to a semicircle, which could suggest that the corrosion process of this coating runs with activation control. The Bode plot also confirmed one a phase angle maximum at 0.4–1.0 Hz and impedance modules |Z|_0.001 Hz_ reaching 40–70 kΩ cm^2^ for this coating (Fig. 12b). Attention should be paid to the visible ‘flattening’ of the spectrum of the sample deposited at − 0.8 V in the range of several mHz (Fig. 12a). This could indicate, e.g. some role of diffusion limitations in the corrosion process. The same behavior was observed for the impedance spectra (not presented here) recorded for the coatings deposited at − 0.7 and − 0.9 V.

**Figure 13:**
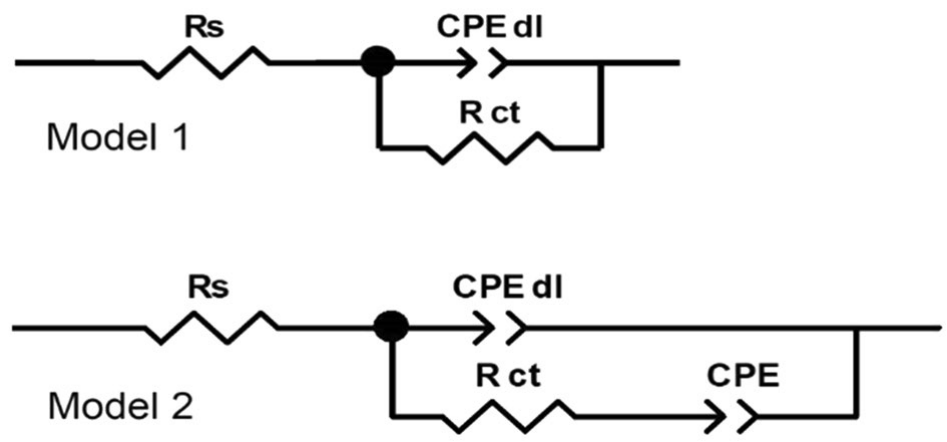
Electric equivalent circuits used for fitting the impedance spectra recorded after 18 h of immersion of Ni–Mo alloy coatings in a 0.05 mol dm^−3^ NaCl solution.

Model 1 presented in Fig. 13 was used for the initial investigation. The physical sense of its electric elements was as follows: R_s_ is the resistance of NaCl electrolytic solution, R_ct_ is the charge transfer resistance—metal oxidation, CPE_dl_ is the constant phase element, characterized by T and P parameters, indirectly related to the double-layer capacitance. The adopted model gave a good fit to the experimental spectra, low values of χ^2^ parameter (~ 10^–4^) and low residual errors: 0.1–11%, but not in the full frequency range, i.e. the single mHz range was cut off.

Unfortunately, this model did not work properly for coatings deposited at − 0.7 V, − 0.8 V, and − 0.9 V, so in their case, a modified circuit (‘Model 2’ in Fig. 13) was used in which the second constant phase element was added in series with R_ct_. This approach allowed one to simulate the flattening of the low-frequency portion of the spectrum and avoid the use of a Warburg element for which, due to the shape of the spectrum, it was not possible to assume the parameter W_s_–P = 0.5. The fitting results are presented in Table 2.

**Table 2:** Fitting results for impedance spectra recorded after 18 h immersion in 0.05 mol dm^−3^ NaCl solution of Ni–Mo coatings calculated using both electric equivalent circuits presented in Fig. 13.

Deposition potential (V vs. Ag)	R_s_ (Ω cm^2^)	CPE_dl_-T (Ω^−1^ cm^−2^ s^P^)	CPE_dl_-P	R_ct_ (kΩ cm^2^)	CPE-T (Ω^−1^ cm^−2^ s^P^)	CPE-P
− 0.5 V	80	2.0 × 10^−4^	0.93	57.1	…^a^	…^a^
− 0.6 V	90	1.5 × 10^−4^	0.89	67.1	…^a^	…^a^
− 0.7 V	103	1.6 × 10^−4^	0.88	11.5	1.4 × 10^−4^	0.32
− 0.8 V	59	1.2 × 10^−4^	0.90	21.8	7.4 × 10^−5^	0.26
− 0.9 V	69	3.3 × 10^−4^	0.89	25.1	1.3 × 10^−4^	0.25^b^

^a^For coatings deposited at − 0.5 V and − 0.6 V Model 1 from Fig. 13 was used for the fitting procedure, while for these deposited at − 0.7 V, − 0.8 V and − 0.9 V—‘Model 2’ was adopted.

^b^The parameter P of CPE was fixed to 0.25.

According to the results in Table 2, the charge transfer resistance—R_ct_, was the highest, 57–67 kΩ cm^2^, for the coatings deposited at the less negative potentials. The increase in the deposition potential towards more negative values was unfavorable. Undoubtedly, this may be related to the deterioration of the quality of the coatings, as evidenced by SEM microphotographs in Fig. 3. Additionally, it may also be a result of the higher content of oxidized compounds in both the bulk of the coatings and on its surface. However, no significant changes were observed in the parameters T and P characterize the CPE_dl_ element (Table 2).

## Conclusions

Nanocrystalline Ni–Mo alloy coatings were deposited from the DES plating bath based on choline chloride and propylene glycol in a 1:2 molar ratio + 0.2 mol dm^−3^ NiCl_2_·6H_2_O and 0.01 mol dm^−3^ (NH_4_)_6_Mo_7_O_24_·4H_2_O. It has been shown that ethylene glycol can be replaced with less harmful propylene glycol, which undoubtedly has a positive effect on reducing the harmfulness of the entire plating bath. The co-deposition of molybdenum with nickel significantly modified the cathodic process and caused an increase in the cathode current. Furthermore, by changing the deposition potential from − 0.5 to − 0.9 V vs. Ag, the deposition current density increased from − 0.4 to − 1.5 mA cm^−2^, respectively. This trend was accompanied by an increase in the Mo content from ~ 7 to ~ 13 wt% in the potential range from − 0.5 to − 0.7 V. However, a further change of potential from − 0.8 to − 0.9 V caused a decrease in the Mo content to ~ 10 wt% and deterioration of the coating quality.

Uniform and adherent Ni–Mo deposits with a nodular morphology were obtained at all the deposition potentials investigated (from − 0.5 to − 0.9 V vs. Ag). However, the more electronegative potential was, the roughness of the surface increased, as evidenced by the change in the R_a_ parameter, from 0.1 to 0.41 μm. The most uniform coating deposited at − 0.6 V, with a thickness of ca. 660 nm, was characterized by crystallite size that did not exceed 10 nm. With the content of Ni (89 at.%) and Mo (11 at.%), the SAED analysis allowed for the identification of the cubic phase Ni_3.64_Mo_0.36_ in this coating. At the coating/copper base material, TEM analysis revealed the presence of nanometric spherical objects. It was estimated that they are less dense areas than the coating, and more precisely, they are empty spaces (pores) with a diameter between 10 and 40 nm.

The corrosion resistance of Ni–Mo coatings in 0.05 mol dm^−3^ NaCl generally increased during 18 h exposure, as evidenced by the ever-higher polarization resistance. Finally, regardless of the applied deposition potential, low corrosion currents (in the range of 0.1–0.3 μA cm^−2^) have been measured for the coatings. Impedance measurements revealed that the charge transfer resistances were the highest (57–67 kΩ cm^2^) for the coatings deposited at less negative potentials, namely − 0.5 V to − 0.7 V. An increase in the deposition potential in the negative direction was concluded to be unfavorable in terms of the corrosion resistance.

The exploratory research carried out shows the need to increase the deposition rate, which should result in the obtaining thicker coatings. Another goal will be to control a given molybdenum content in Ni–Mo coating. This can be achieved, e.g. by modifying the composition of the electroplating bath.

## Data Availability

The data sets used and/or analyzed during the current study are available from the corresponding author on reasonable request.
